# Molecular Characterization and Protective Efficacy of a Novel Protein (EnSSB) Containing a Single-Stranded DNA-Binding Domain from *Eimeria necatrix*

**DOI:** 10.3390/ani15172482

**Published:** 2025-08-23

**Authors:** Yu Zhu, Dandan Liu, Lele Wang, Qianqian Feng, Nianyu Xue, Zhaofeng Hou, Jinjun Xu, Jianping Tao

**Affiliations:** 1 College of Veterinary Medicine, Yangzhou University, Yangzhou 225009, China; zy18362822874@163.com (Y.Z.); ddliu@yzu.edu.cn (D.L.); 18252736323@163.com (L.W.); qianqianfeng98@163.com (Q.F.); yznianyuxue@126.com (N.X.); zfhou@yzu.edu.cn (Z.H.); jjxu@yzu.edu.cn (J.X.); 2 Jiangsu Co-Innovation Center for Prevention and Control of Important Animal Infectious Diseases and Zoonoses, Yangzhou University, Yangzhou 225009, China

**Keywords:** *Eimeria necatrix*, *SSB* gene, characterization, localization, protective efficacy

## Abstract

SSB proteins play essential roles in DNA replication, recombination, and repair in bacteria, archaea, and eukarya. This study investigates the transcript levels, identification, expression and purification, subcellular localization, and immune protective potential of the SSB-like proteins of *Eimeria necatrix* (EnSSB), exploring its role in the development of *E. necatrix* and its potential as a candidate antigen for a subunit vaccine against avian coccidiosis. The level of EnSSB gene transcription was highest in unsporulated oocysts (UO), followed by gametocytes (GAM) (*p* < 0.05). The gene consisted of an open reading frame of 1488 nucleotides encoding a protein of 495 amino acid residues with a predicted molecular weight of 53.31 kDa. EnSSB contained a SSB domain with a conserved OB (oligonucleotide/oligosaccharide binding) fold. The molecular mass of the native protein, as determined by Western blot analysis, was ~58 kDa in second-generation merozoites (MZ-2) and UO. In addition to the 58 kDa band, four other bands (~98 kDa, ~82 kDa, ~36 kDa and ~28 kDa) were detected in GAM. No bands were detected in MZ-3. Indirect immunofluorescence and immuno-electron microscopy localized EnSSB in the cytoplasm of macrogametocytes but not in wall-forming bodies and oocyst wall. Animal challenge experiments demonstrated that rEnSSB elicited robust IgY responses, increased splenic T lymphocytes and body weight gain, reduced intestinal lesion scores and oocyst shedding, and presented anticoccidial index (ACI) more than 160. These findings not only offer a foundation for understanding the role of EnSSB protein in regulating the development of *E. necatrix*, but also present a potential protective antigen of *E. necatrix* for the development of a subunit vaccine against avian coccidiosis.

## Simple Summary

Coccidiosis is a critical protozoan infection in poultry, induced by parasites belonging to the genus *Eimeria*. Single-stranded DNA-binding protein (SSB) is indispensable elements in cells of all living organisms. However, to date, no SSB gene was identified and characterized in *Eimeria* species. *ENH_00003220* gene of *Eimeria necatrix* is annotated as a hypothetical protein, which contains a single-stranded DNA-binding domain. In this study, the transcript level of this gene (EnSSB) was analyzed using *q*PCR. EnSSB gene was cloned and expressed. The native protein of EnSSB and its subcellular localization was analyzed. The immune protection provided by recombinant EnSSB (rEnSSB) was evaluated in chicken. Results showed that EnSSB existed in *E. necatrix* with a molecular weight of ~58 kDa, and was localized in the cytoplasm of macrogametocytes; rEnSSB provided effective protection against challenge infection with *E. necatrix*.

## 1. Introduction

Coccidiosis in chickens is an intestinal disease caused by intracellular protozoan parasites of the genus *Eimeria* [1]. It poses a serious threat to the poultry industry [2,3], causing an estimated annual economic loss of approximately £10.4 billion worldwide [4]. The seven *Eimeria* species are known to parasitize chickens, among which *Eimeria necatrix* (*E. necatrix*) exhibits the highest pathogenicity [5]. *E. necatrix* mainly infects chickens aged 8 to 18 weeks, inducing acute small intestinal coccidiosis that result in high morbidity and mortality within affected flocks [6,7]. Current control of coccidiosis is accomplished through effective husbandry practices, the use of anticoccidial medications, and/or vaccination with live parasites [5,8]. While pharmaceutical prevention is both cost-effective and successful, the emergence of drug resistance and public demand for residue-free meat constrain its widespread use [9]. Furthermore, the widespread use of live anticoccidial vaccines in the poultry industry is hindered by the potential risk of vaccinal pathogenicity and the high production costs [10,11]. Recombinant vaccines have demonstrated potential as an effective method for controlling coccidiosis. Therefore, research focused on identifying novel and potent *Eimeria* antigens is crucial for the development of recombinant vaccines against this disease.

Like other apicomplexan parasites, *Eimeria* parasites encompass three principal stages involving merogony (asexual reproduction), gametogony (sexual reproduction), and sporogony. During gametogony, macrogametocytes and microgametes fuse to form zygotes, which then developed into oocysts, with an oocyst wall forming around them. Unsporulated oocysts are shed in feces and undergo sporulation in the external environment, thereby generating infectious, sporulated oocysts that facilitate disease transmission between hosts [12,13]. It is evident that the differentiation from one developmental stage to the next is crucial for the survival, reproduction, and transmission of Eimeria parasites. However, the mechanisms underlying this transition between stages remain incompletely understood. Therefore, elucidation of the key proteins involved during the development and differentiation is essential for uncovering new targets for the prevention and treatment of avian coccidiosis.

In our previous work, we employed next-generation sequencing to generate both transcriptomic and proteomic datasets for *E. necatrix* at four developmental stages: second-generation merozoites (MZ-2), third-generation merozoites (MZ-3), gametocytes (GAM) and unsporulated oocysts (UO), and two types of structural components: wall-forming bodies and oocyst wall. Comparative analysis of these data revealed differentially expressed genes across developmental stages, among which the gene (NCBI: *ENH_00003220*) exhibited negligible expression in MZ-2 and MZ-3 but was highly upregulated in GAM and UO [14,15,16,17,18]. So far, the *ENH_00003220* gene is annotated as a hypothetical protein (NCBI: XP_013433731.1). However, with the conserved domain analysis of the amino acid sequence encoded by the gene, we found that *ENH_00003220* gene contains a single-stranded DNA-binding domain.

Single-stranded DNA-binding protein (SSB), also known as replication protein A (PRA) in eukaryotes [19,20], was first isolated from T4 bacteriophage-infected cells of *Escherichia coli* [21]. It was subsequently found in eukaryotes, bacteria, and archaea [22,23], and they are also encoded by bacteriophage and adenovirus genomes [24,25]. A hallmark of the SSB protein family is their high-affinity, sequence-independent binding to single-stranded DNA (ssDNA). Beyond their canonical role in ssDNA stabilization, SSB proteins participate in maintaining genome integrity and orchestrating essential cellular processes, including DNA replication, recombination, and repair, thereby serving as pivotal factors throughout the cellular life cycle [26]. In solution, SSB proteins exist in various oligomeric forms, including homodimers (SSBs from bacteriophages, *Thermus thermophilus*, *Thermus aquaticus* and *Deinococcus radiodurans*), homotetramers (mitochondrial, crenarchaeal and most prokaryotic SSBs) and heterotrimers (euryarchaeal and eukaryotic SSBs, alias RPAs) [27,28]. To date, SSB proteins or PRA in protozoan have been identified from *Crithidia fasciculata* [29], *Cryptosporidium parvum* [30,31] and *Plasmodium falciparum* [32]. However, no SSB or RPA gene was identified and characterized in *Eimeria* species.

In the current study, the *ENH_00003220* gene (EnSSB) was cloned and expressed in *Escherichia coli* BL21 (DE3) using pET28a (+) plasmid as the expression vector. The native EnSSB protein and its subcellular localization in *E. necatrix* were analyzed through Western blotting, indirect immunofluorescence assay (IFA) and the immunogold electron microscopic co-localization technique, respectively. Transcript levels of EnSSB in different developmental stages of *E. necatrix* was analyzed using qPCR. Ultimately, a vaccination and challenge trial was performed to evaluate the immune protection induced by the rEnSSB protein in chickens. Together, these results establish a foundation for understanding the regulatory role of the EnSSB protein in *E. necatrix* development and could facilitate the development of a recombinant subunit vaccine against avian coccidiosis.

## 2. Materials and Methods

### 2.1. Parasite, Animal and Antibody

*Eimeria necatrix* (Yangzhou strain) employed in this study was originally isolated in 2009 from chickens that succumbed to *E. necatrix* infection in Yangzhou, China, with identification confirmed through morphological and molecular analyses [33]. The oocysts of *E. necatrix* were serially passaged in 3–4-week-old chickens. Oocysts were isolated from fecal samples using a salt flotation method followed by centrifugation. The oocysts were then sporulated in vitro at 28 °C and stored in a 2.5% potassium dichromate solution at 4 °C [33].

A total of 200 one-day-old chickens were obtained at day 1 of age from the Poultry Institute of Chinese Academy of Agricultural Sciences (Yangzhou, China). The chickens were kept in a coccidium-free environment and provided water and food ad libitum, without the inclusion of anticoccidial agents or antibiotics. Chickens were all euthanized by inhalation anesthesia using ether after the experiment. Six-week-old specific-pathogen-free (SPF) female BALB/c mice were obtained from Yangzhou University and housed under SPF conditions. After the experiment, 10 mice were all euthanized using carbon dioxide. All animal handling and procedures were performed in accordance with the guidelines for animal use in toxicology. The study protocols received approval from the Animal Care and Use Committee of the College of Veterinary Medicine, Yangzhou University (Approval ID: SYXK [Su] 2021-0027, Date: 26 March 2021).

*E. necatrix* gametocyte proteins 22 and 59 (EnGAM22 and EnGAM59) are localized to types 1 and 2 wall-forming bodies (WFB1 and WFB2) in macrogametocyte and involved in the formation of the outer and inner layers of the oocyst wall (OW and IW) of *E. necatrix*, respectively [34,35]. Rat polyclonal antibody against recombinant EnGAM22 (rat anti-rEnGAM22 pAb) and rat polyclonal antibody against recombinant EnGAM59 (rat anti-rEnGAM59 pAb) were produced by immunization with rEnGAM22 protein or rEnGAM59 protein expressed in a eukaryotic system in our laboratory.

### 2.2. Preparation of Parasites in Different Developmental Stages of E. necatrix

In order to collect second-generation merozoites (MZ-2), third-generation merozoites (MZ-3), gametocytes (GAM) and unsporulated oocysts (UO), thirty 20-day-old chickens were orally infected with approximately 1.7 × 10^4^ sporulated oocysts of *E. necatrix*. MZ-2 and MZ-3 were collected and prepared according to previously described methods [36]. Briefly, the intestinal tissues were collected at 138 h post-infection (hpi) and processed for merozoite isolation. The obtained MZ-2 were purified by Percoll density gradient centrifugation. Cecum tissues were collected at 150 hpi and processed for merozoite isolation and the obtained MZ-3 were purified by DEAE-52 cellulose column chromatography. GAM was collected and prepared using previously described methods [33]. In brief, cecum tissues were collected at 154 h post-infection (hpi), digested with 0.5 mg/mL hyaluronidase, and then sequentially filtered through 17 μm and 10 μm polymer meshes (Sefar Filtration Solution Co., Ltd., Suzhou, China). Subsequently, the GAM was rinsed from the mesh using cold SAC solution and centrifuged at 1000× *g* for 5 min. UO was then isolated and purified using a saturated salt and sucrose solution [15].

### 2.3. Transcript Levels of EnSSB in Different Developmental Stages of E. necatrix

Total RNA of MZ-2, MZ-3, GAM and UO strains of *E. necatrix* were isolated using the RNA Extraction Kit™ (TaKaRa, Tokyo, Japan). The cDNA synthesis was generated with the PrimeScript RT reagent Kit with gDNA Eraser (TaKaRa Bio. Inc., Shiga, Japan). Specific primers were designed to amplify the EnSSB cDNA for qPCR (Table 1) analysis. The 18 S ribosomal RNA of *E. necatrix* (En18S rRNA) served as the internal reference. qPCR was conducted with the AceQ^®^ qPCR SYBR Green Master Mix (Vazyme, Nanjing, China) following manufacturer’s protocol. Each sample was tested in biological triplicate, and the experiment was repeated three times. The relative expression of EnSSB mRNA was quantified using the 2^−ΔΔCt^ method [37]. Statistical significance was determined at *p* < 0.05.

### 2.4. Cloning and Sequence Analysis of EnSSB

The total RNA of GAM was isolated using RNA Extraction Kit™ (TaKaRa, Tokyo, Japan), followed by reverse transcription into cDNA using the Reverse Transcriptase Kit™ (TaKaRa, Tokyo, Japan). The target cDNA was amplified using the RNA LA PCR Kit™ (TaKaRa Bio. Inc., Shiga, Japan) following manufacturer’s protocol with a pair of specific primers (Table 1) designed based on a novel sequence (GenBank accession number: *ENH_00003220*). The PCR products were analyzed by electrophoresis on a 1.0% agarose gel.

The RT-PCR products were subcloned into the pGEM^®^-T-easy vector (Promega, Madison, WI, USA) and sequenced by a commercial company (Beijing Genomics institution, Beijing, China). The sequencing results were analyzed with BLASTN, the protein sequences were predicted with DNAStar software Lasergene 7.0, and the conserved-domains were predicted with NCBI webpage (http://www.ncbi.nlm.nih.gov/, accessed on 17 January 2025). The tertiary structure of the protein and its conserved domain were predicted using the Swiss-model tool (https://swissmodel.expasy.org/interactive, accessed on 17 January 2025). The schematic representation of the secondary structure of EnSSB OB-flod was predicted with ESPript 3.0 (https://espript.ibcp.fr/ESPript/cgi-bin/ESPript.cgi, accessed on 17 January 2025). The subcellular localization was predicted using Uniport (https://www.uniprot.org/uniprotkb/U6MT59/entry, accessed on 17 January 2025). A phylogenetic tree was constructed using the neighbor-joining method with 1000 bootstrap replicates in MEGA software (version 5.05, State College, PA, USA) (http://www.megasoftware.net/, accessed on 17 January 2025).

### 2.5. Expression and Purification of the Recombinant EnSSB Protein

According to the sequencing data, specific primers containing *Nde* I and *Bam*H I restriction sites were designed to amplify the EnSSB encoding sequence, excluding the signal peptide. The product was inserted into pET28a (+) expression vector (Invitrogen), sequenced, and transformed into chemically competent *Escherichia coli* BL21 (DE3) cells (Invitrogen) for further protein expression. The recombinant EnSSB (rEnSSB) was expressed and purified as described previously [33]. Briefly, the recombinant protein expression in *E. coli* was induced by adding 1.0 mM isopropyl β-D-1-thiogalactopyranoside (IPTG; Promega Corp.) to the cell culture when the culture reached an OD_600_ of 0.6, followed by incubation at 37 for 4 h. Cells were harvested, lysed, and analyzed by 12% sodium dodecyl sulfate polyacrylamide gel electrophoresis (SDS-PAGE), which was visualized after staining with Coomassie brilliant blue. The recombinant proteins were purified using a Ni^2+^-nitrilotriacetic acid (Ni-NTA) column (GE Healthcare, Saint Louis, MO, USA) according to the manufacturer’s instructions. Purified recombinant proteins were refolded by renaturation buffer (50 mM Tris–HCl, 0.15 M NaCl, pH 8.0) containing different concentrations of urea (6, 4, 2, 1, 0 mol/L), and then concentrated using polyethylene glycol (PEG8000). The rEnSSB yield was estimated by measuring absorbance at 280 nm using the NanoDrop2000 spectrophotometer (Thermo Fisher Scientific, Saint Louis, MO, USA). The purified recombinant proteins were visualized on 12% SDS-PAGE, aliquoted, and stored at −20 °C until further use.

### 2.6. Preparation of Mouse Polyclonal Antibodies Against rEnSSB

Mouse polyclonal antibody against rEnSSB (mouse anti-rEnSSB pAb) were prepared. In brief, the 50 μg of proteins were resuspended in 50 μL phosphate-buffered saline (PBS) and mixed with 50 μL Quick Antibody-Mouse 3W (Biodragon, Beijing, China). This mixture was used to immunize 6-week-old BALB/c mice twice, following the manufacturer’s instructions. Blood samples were collected 7 days after the second immunization, centrifuged at 1500× *g* for 15 min to isolate the mouse polyclonal antibodies (pAbs). Antibody levels were measured using an enzyme-linked immunosorbent assay (ELISA) as described in a previous study [33]. The results indicated that the optical density (OD) value of the mouse anti-rEnSSB pAb was 2.36 (1:200 dilution).

### 2.7. Immunoblotting Analysis for rEnSSB and Native EnSSB in Different Developmental Stages of E. necatrix

To identify the recombinant protein, *E. coli* cell lysates were subjected to 12% SDS-PAGE, followed by transfer to a nitrocellulose membrane (Merck Millipore, Billerica, MA, USA). The membranes were blocked with 3% bovine serum albumin (BSA) in tris-buffered saline (TBS) overnight at 4 °C. Subsequently, they were incubated with anti-6×His epitope tag monoclonal antibody (dilution, 1:2000, Beyotime, Shanghai, China) for 1 h at 37 °C, followed by three washes with 0.03% Tween-20/TBS (TBST) for 10 min each. The membrane-bound antibodies were then detected using HRP-labeled rabbit anti-mouse IgG (1:20,000 dilution, BIO BASIC, Markham, ON, Canada) for 45 min at 37 °C. After washing with TBST, the membranes were visualized with the Hypersensitive ECL Chemiluminescence Kit (Tanon, Shanghai, China) for 1 min in a darkroom. Images were captured using the Tanon-5200 Chemiluminescent Imaging System.

To confirm the presence of the native EnSSB protein, the protein extracts from MZ-2, MZ-3, GAM and UO were prepared according to the method described by Su et al. [17,18], and then were detected by Western blot analysis using anti-rEnSSB pAb with above methods.

### 2.8. Subcellular Localization of EnSSB Protein in E. necatrix Macrogametocytes

#### 2.8.1. Indirect Immunofluorescence Assay

Tissue sample preparation and indirect immunofluorescence analysis (IFA) were conducted as described previously [33]. In brief, ten 20-day-old chickens were orally infected with 3.8 × 10^4^ *E. necatrix* sporulated oocysts and euthanized at 148 hpi. The caeca were excised and rinsed with cold PBS. The cecum tissues were immediately fixed in 3% paraformaldehyde in PBS. After fixation, the tissues were embedded in paraffin and sectioned into 4 μm-thick slices using a microtome at room temperature. The paraffin was removed from the sections, followed by inactivation of endogenous enzymes with 3% H_2_O_2_ and antigen retrieval with 0.1% trypsin (Promega Corp.). The sections were then blocked overnight with 5% BSA in PBS at 4 °C, the sections were incubated with mouse anti-rEnSSB pAb (1:50 dilution) and rat anti-rEnGAM22 pAb or rat anti-rEnGAM59 pAb (1:50 dilution) for 1 h at 37 °C. After incubation, the sections were washed three times for 15 min each with 0.03% Tween-20/PBS (PBST). Subsequently, the sections were incubated with fluorescein isothiocyanate (FITC)-conjugated goat anti-rat IgG (1:100 dilution; MultiSciences Hangzhou, China) and AF647-conjugated goat anti-mouse IgG (1:100 dilution; Servicebio) with 5% BSA in PBS for 1 h at 37 °C. After incubation, the samples were rinsed with PBST and counterstained with 4′6-diamidino-2-phenylindole (DAPI, Biyotime, Shanghai, China). Images were captured using LCM (Leica TCS SP8 STED, Wetzlar, Germany). Portions of the same paraffin-embedded tissue samples were also stained with hematoxylin and eosin to verify the development of gametocyte and oocyst in caecum mucosal tissues.

#### 2.8.2. Immunogold Electron Microscopic Co-Localization

Preparation of tissue samples and immunogold electron microscopic co-localization were performed as our previously described [35]. Briefly, the pathological cecum samples after cleaning with cold PBS were fixed in the specialized fixation solution for immunoelectron microscopy (IEM), and then cut into approximately 1 mm^3^ blocks using a surgical blade. These tissue blocks were fixed with fresh fixation solution at 4 °C, and then dehydrated through a graded ethanol series (30%, 50%, 70%, 80%, 85%, 90%, 95%, and 100%) before embedding in resin. After polymerizing in low temperature UV polymerizer (Electron Microscopy, Beijing, China) for approximately 48 h at −20 °C, the resin blocks were sectioned to a thickness of 70–80 nm using an ultramicrotome (Leica UC7, Wetzlar, Germany). The ultrathin sections were then placed onto 150-mesh nickel grids with formvar film. The nickel grids with sections were submerged in ultrapure water for 5 min, followed by three 5 min rinses with TBS. After the rinsing steps, the sections were blocked with 1% BSA in TBS for 30 min at room temperature, and then were incubated with mouse anti-rEnSSB pAb (1:200 dilution) and rat anti-rEnGAM22 pAb (1:200 dilution) overnight at 4 °C. After washing with TBS three times for 15 min each time, the sections were incubated with 10 nm gold particle-labeled goat anti-mouse secondary antibody (Sigma-Aldrich, St. Louis, MO, USA) and 18 nm gold particle-labeled goat anti-rat secondary antibody (Sigma-Aldrich, St. Louis, MO, USA) for 20 min at room temperature, followed by 1 h at 37 °C, and then 0.5 h at room temperature. The sections were subsequently examined using transmission electron microscopy (TEM) (HT7800, Hitachi, Tokyo, Japan).

### 2.9. Evaluation of Immune Protection

#### 2.9.1. Immunization Trail

Referring to published research [28,38], the immunization procedure and experimental design were presented in Table 2. In total, 160 chickens with similar weight were randomly divided into eight groups (n = 20, for each) at 6 days of age. The chickens in groups 1 and 2 (IC-H-1, IC-H-2), 3 and 4 (IC-M-1, IC-M-2), 5 and 6 (IC-L-1, IC-L-2) were immunized with high-dose (200 μg), middle-dose (100 μg), and low-dose (50 μg) of rEnSSB protein, respectively. The chickens in groups 7 and 8 were served as the positive (unimmunized and challenged with oocysts group, UC) and negative (unimmunized and unchallenged with oocysts group, UU) controls, respectively. On day 6, chickens in groups 1–6 received the initial immunization of rEnSSB protein via subcutaneous injection in the thighs, while the control groups were injected with PBS. A booster injection was administered on day 13. On day 20, chickens in groups 1–6, along with the UC group, were challenged with an oral inoculation of 3 × 10^4^ sporulated oocysts of the *E. necatrix* Yangzhou strain. On day 28 (8 days post-challenge), the chickens were euthanized, and the small intestines were collected for lesion examination.

In addition, seven days after the primary and booster immunizations, five chickens from each group were euthanized, and their spleens were harvested to assess CD4^+^ and CD8^+^ T cells populations. Concurrently, blood sera were collected from the chickens to measure specific antibody levels.

#### 2.9.2. Evaluation of the Protective Efficacy of rEnSSB Against Challenge Infection with *E. necatrix*

The efficacy of immunization was assessed based on survival rate (SR), body weight gain (BWG), reduction in oocyst production (ROP), lesion score (LS), and anticoccidial index (ACI). SR (%) is calculated using the formula: (the number surviving per group/the number of initial chickens) × 100%. BWG of chickens in each group was calculated from days 20–28 in challenge period. The feces of each group were collected every 12 h on days 6 to 8 post-infection, and cecal contents of each group were collected at the end of the test. The oocysts in feces and cecal contents were determined using McMaster’s counting technique. The total oocyst production (OP) per bird from feces and ceca was used to calculate ROP using the formula: ((oocyst output of UC group − oocyst output of IC groups)/oocyst output of UC group) × 100%. On day 8 post-challenge, all the chickens were euthanized, and the intestinal lesions were scored on a scale of 0 to 4, based on the method described by Johnson and Reid [39].

ACI value is calculated with the formula: (SR + RBWG (%) − (LI (lesion index) + OI (oocyst index)) according to the previous description [40]. RBWG (%) is calculated using the formula: (BWG of IC or UC groups/BWG of UU group) × 100%. LI is calculated using the formula: average LS is multiplied by 10. OI is based on the ratio of oocyst output of IC groups to oocyst output of UC group. The ratio is divided into 0~1%, 1~25%, 26~50%, 51~75% and 76~100% five grades, and the corresponding OI is recorded as 0, 5, 10, 20 and 40. ACI value of ≥180 was considered high performance, An ACI value between 160 and 179 was considered effective, while an ACI value of less than 160 was deemed ineffective [40].

#### 2.9.3. Detection of Serum Antibody Levels Against rEnSSB Using ELISA

Blood sera collected above were used to measure the antibody (IgY) response to rEnSSB using indirect ELISA according to previously described methods [38]. Circulating IgY levels were determined through ELISA after laboratory standardization of antigen and serum concentrations via checkerboard titration. For the ELISA, an antigen concentration of 1 μg/well, a serum (1:200 dilution), and an HRP-conjugated goat anti-chicken IgY (H + L) antibody (1:10,000 dilution) were applied. All samples were analyzed in three times.

#### 2.9.4. Assessment of rEnSSB-Induced Alterations in CD4^+^ and CD8^+^ T Cells Using Flow Cytometry

Five chickens from each group were euthanized one week after the primary and booster immunization. Spleens were harvested, and splenic lymphocytes were isolated using a separation solution (Solarbio, Beijing, China). The isolated splenic lymphocytes (1 × 10^6^ cells/mL) were then dual-stained with anti-CD3^+^-FITC antibody and anti-CD4^+^-PE antibody, or anti-CD3^+^-FITC antibody and anti-CD8^+^-PE antibody (Southern Biotech, Birmingham, AL, USA). Finally, the cells were analyzed using a BD FACSMelody flow cytometer (Franklin Lakes, New Jersey, NY, USA).

### 2.10. Statistical Analysis

The statistical significances between the means of different treatment groups were analyzed using one-way analysis of variance (ANOVA) followed by Duncan’s multiple range tests, using SPSS software (IBM SPSS Statistics 20 version, Chicago, IL, USA). Significant differences were indicated as * *p* < 0.05, ** *p* < 0.01, *** *p* < 0.001, and **** *p* < 0.0001.

## 3. Results

### 3.1. Transcript Levels of EnSSB Gene

**Figure 1:**
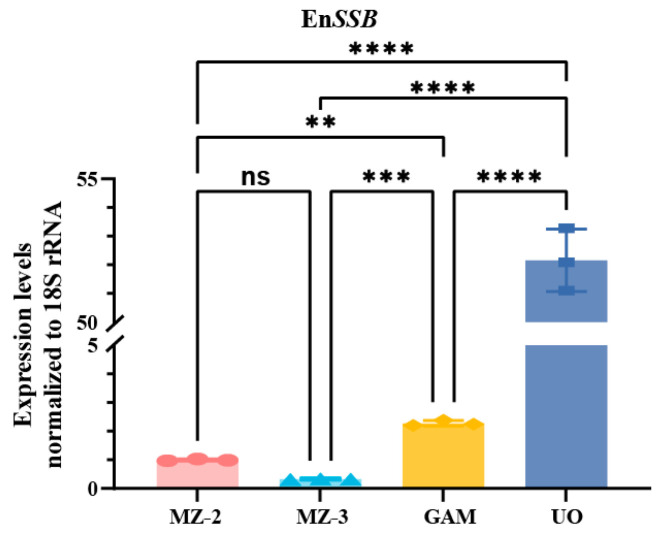
The transcriptional levels of EnSSB at different developmental stages of *E. necatrix*. The transcriptional levels of EnSSB at MZ-2, MZ-3, GAM and UO (n = 3); MZ-2: The second generation merozoites; MZ-3: The third generation merozoites; GAM: gametocytes; UO: unsporulated oocysts. ns *p* > 0.05, ** *p* < 0.01, *** *p* < 0.001, **** *p* < 0.0001.

The transcript levels of EnSSB at various developmental stages of *E. necatrix* were assessed by qPCR (Figure 1). The EnSSB transcript level in UO was significantly higher than that in MZ-2, MZ-3 and GAM (*p* < 0.001). The EnSSB transcript level in GAM was higher than that in MZ-2 and MZ-3 (*p* < 0.01), while no significant difference was observed between MZ-2 and MZ-3 (*p* > 0.05).

### 3.2. Cloning and Sequence Analysis of EnSSB Gene

**Figure 2:**
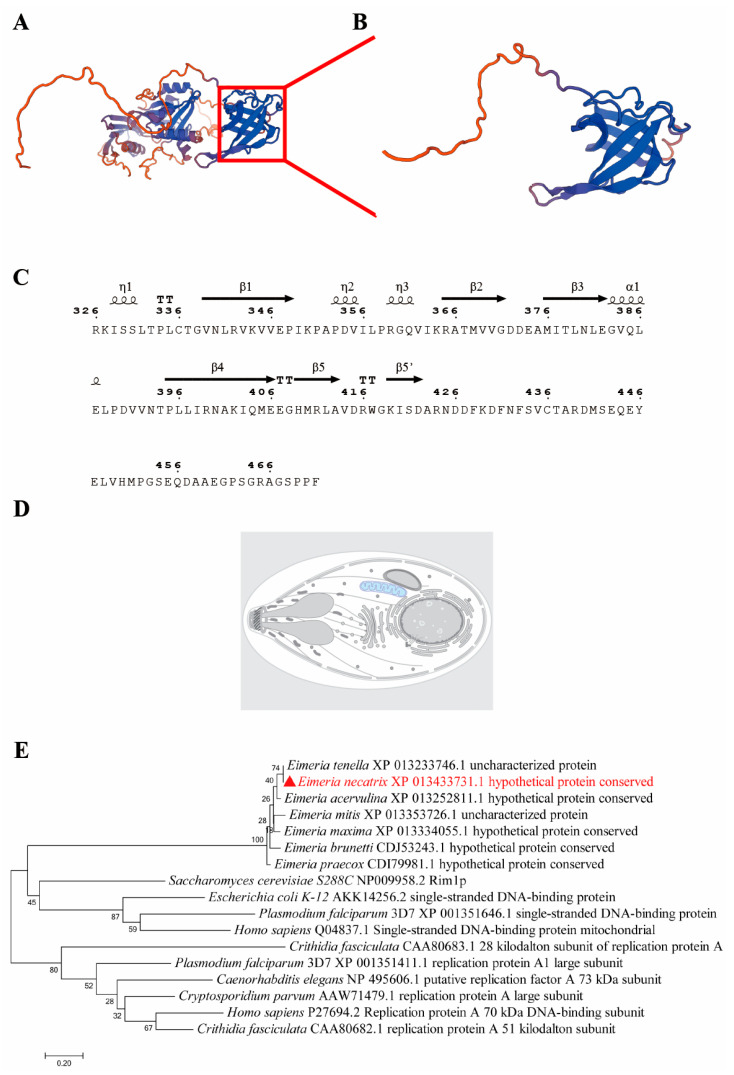
Characteristics of the EnSSB gene. (**A**) The prediction of tertiary structure of EnSSB; (**B**) The prediction of tertiary structure of SSB domain of EnSSB; (**C**) The schematic representation of the secondary structure of EnSSB OB-flod; (**D**) The prediction of subcellular localization of EnSSB protein sites; (**E**) Phylogenetic tree of the SSB domain region from EnSSB and its homologous proteins using the neighbor-joining method (NJ). "▲" represents the SSB domain region from EnSSB.

**Figure A1:**
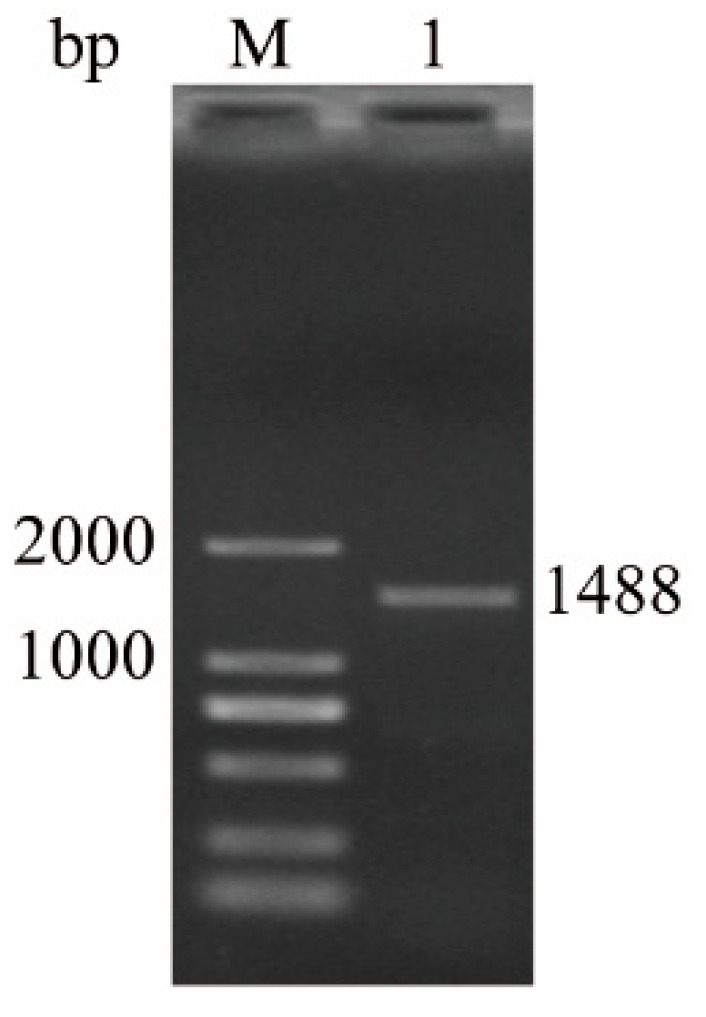
EnSSB PCR products. M: DL2000 DNA Marker; 1: EnSSB PCR products.

The RT-PCR amplification results revealed that the EnSSB cDNA sequence contained 1488 base pairs (bp) (Figure A1). The open reading frame (ORF) of the EnSSB cDNA encoded a 495-amino-acid (aa) protein, which lacked a signal peptide, and with a predicted molecular weight of approximately 53.31 kDa and an isoelectric point of 7.86. The sequence analysis of protein showed that EnSSB has a single-stranded DNA-binding domain located at the C-terminus (326–470 aa), with a 99.9% homology with the sequence of *Eimeria necatrix* Houghton strain (GenBank ID: *ENH_00003220*). The tertiary structure of EnSSB predicted by SWISS-MODEL (Figure 2A), along with the structural model of its conserved domain (Figure 2B), revealed a characteristic OB-fold architecture. The OB-fold domain, predicted with ESPript 3.0, consists of five beta strands (β1–β5) and one alpha helix (α) (Figure 2C). Subcellular localization prediction by UniProt showed that EnSSB has a mitochondrial localization (Figure 2D).

To place EnSSB in the broader evolutionary context of SSB protein family members, the amino acid sequences of SSB from seven species of avian *Eimeria* spp., two apicomplexan parasites (*Cryptosporidium parvum*, *Plasmodium falciparum*), *Escherichia coli*, *Saccharomyces cerevisiae*, *Crithidia fasciculata*, *Caenorhabditis elegan*, *Caenorhabditis elegans* and *Homo sapiens* were used to create an unrooted maximum-likelihood-based phylogenetic tree. As seen in Figure 2E, two branching clades were present. One clade mainly consisted of RPA large subunit (RPA1) of *C. parvum*, *P. falciparum* and human. Another clade was further divided into two branches, one consisting of hypothetical proteins with a single-stranded DNA-binding domain from avian *Eimeria* spp., and the other consisting of SSB proteins from *Escherichia coli*, mitochondria of yeast and human, and apicoplast of *P. falciparum*. EnSSB appeared to be closely related to their homologs in mitochondrial SSB protein and apicoplast SSB protein.

### 3.3. Expression and Purification of rEnSSB

**Figure 3:**
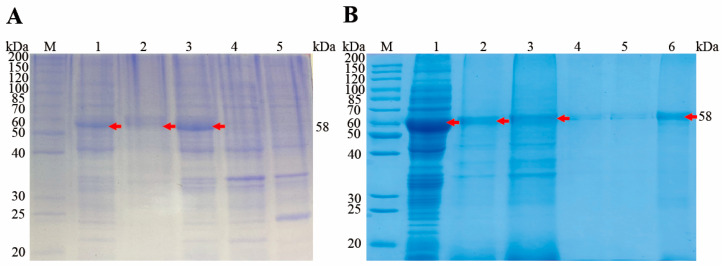
Expression of recombinant protein EnSSB. (**A**) Induction and soluble SDS-PAGE analysis of rEnSSB. Lane M: Unstained protein ladder; Lane 1: Sediments of pET28a (+)-EnSSB /BL21; Lane 2: Supernatant of pET28a (+)-EnSSB /BL21; Lane 3: Bacterial lysates of pET28a (+)-EnSSB /BL21; Lane 4: pET28a (+)/BL21; Lane 5: BL21 with IPTG induction. (**B**) Purification of rEnSSB. Lane M: Unstained protein ladder; Lane 1: Supernatant of inclusion bodies after solubilizing by urea; Lane 2: Effluent after binding to Ni-NTA; Lane 3–5: Wash fractions; Lane 6: Eluted protein from Ni-NTA. Red arrows indicate the band of the EnSSB protein.

A PCR product of 1488 bp was excised from an agarose gel and inserted into the pET28a (+) bacterial expression vector, which contains a 6×His tag at the N-terminus, before transforming it into chemically competent *E. coli* BL21(DE3). After induction with 1.0 mM IPTG for 4 h at 37 °C, the recombinant proteins were analyzed by 12% SDS-PAGE, which revealed a protein band of ~58 kDa after staining with Coomassie brilliant blue (Figure 3A, lane 3). No protein was detected in the negative control (Figure 3A, lane 4, 5). The recombinant protein was expressed in inclusion bodies (Figure 3A, lane 1), and was purified using Ni-NTA chromatography column (Figure 3B). After concentration with PEG8000, the final protein concentration was determined to be 2 mg/mL.

### 3.4. Western Blot Analysis for the Recombinant and Native EnSSB

**Figure 4:**
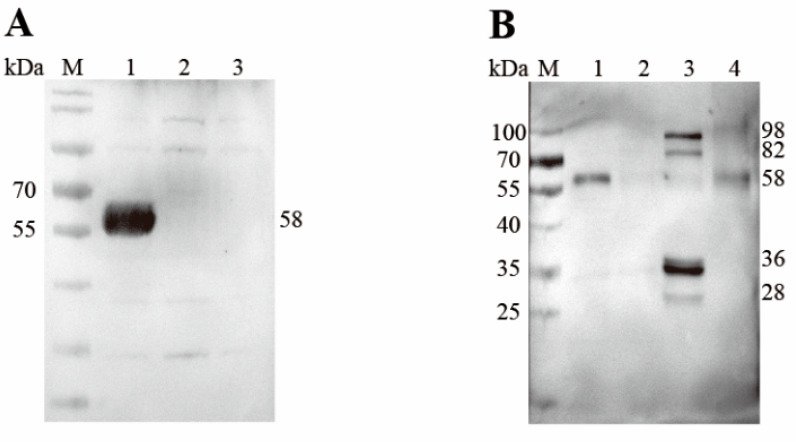
Western blot analysis for the recombinant EnSSB and crude somatic extracts of *E. necatrix*. (**A**) Western blot analysis of rEnSSB. Lane M: Prestained protein ladder; Lane 1: pET28a (+)-EnSSB/BL21 with IPTG induced; Lane 2: pET28a (+)/BL21 with IPTG induced; Lane 3: BL21 with IPTG induced; (**B**) Detection of EnSSB native protein, the primary antibody was anti-rEnSSB pAb; Lane M: Prestained protein ladder; Lane 1: The second generation merozoites (MZ-2); Lane 2: The third generation merozoites (MZ-3); Lane 3: gametocytes (GAM); Lane 4: unsporulated oocysts (UO).

A band of the expected size, 58 kDa, was observed when bacterial lysates containing the recombinant protein were probed with the anti-6×His tag monoclonal antibody (Figure 4A), which indicated that rEnSSB was successfully expressed in vitro. Western blot analysis further revealed that mouse anti-rEnSSB pAb recognized the native EnSSB as a band of approximately 58 kDa (~58 kDa) in the extracts of MZ-2 and UO of *E. necatrix* (Figure 4B), slightly larger than the deduced one. But no band was detected in the extract of MZ-3. In contrast, the extract of GAM showed four distinct bands at ~98, ~82, ~36, and ~28 kDa, along with an extremely weak band at ~58 kDa.

### 3.5. Subcellular Localization of Native EnSSB

**Figure 5:**
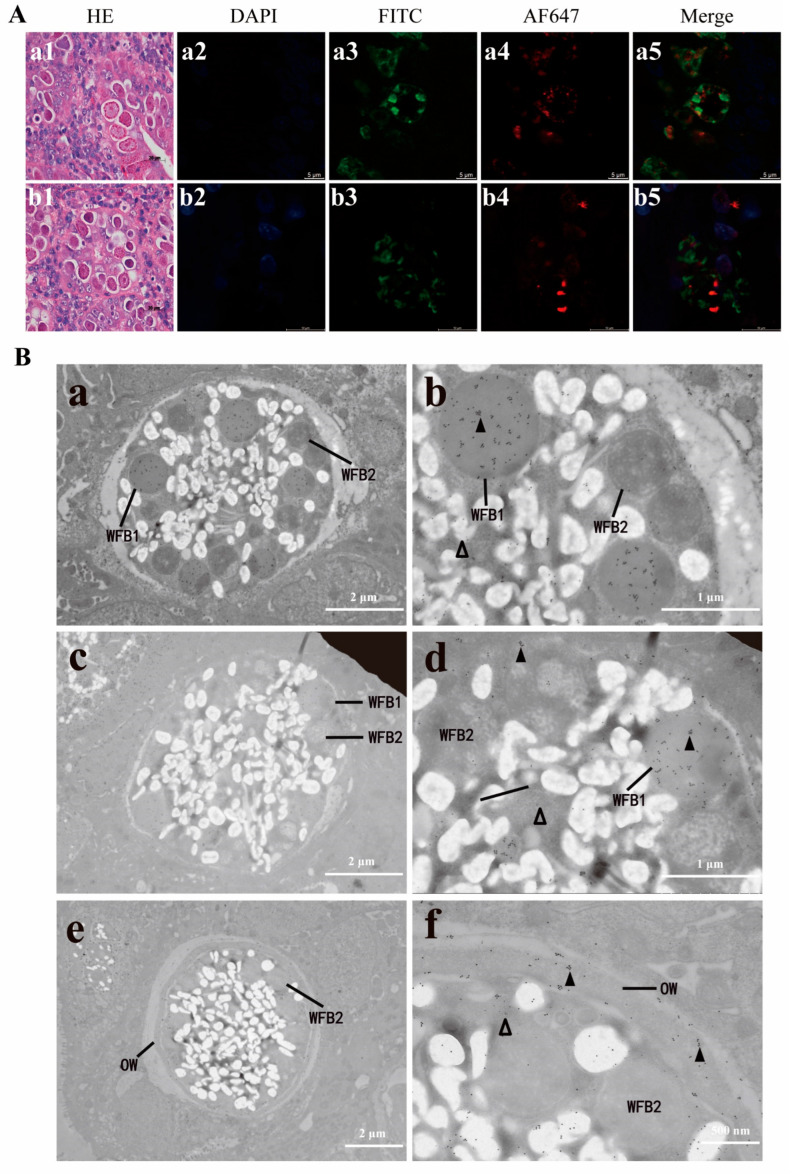
Localization of EnSSB in macrogametocytes of *Eimeria necatrix*. (**A**) The results of indirect immunofluorescence localization of EnSSB. (**a**) Immunolabeled with mouse anti-rEnSSB pAb and rat anti-rEnGAM22 pAb. (**b**) Immunolabeled with mouse anti-rEnSSB pAb and rat anti-rEnGAM59 pAb. HE: Paraffin sections stained with HE; DAPI: Counter-stained with DAPI; FITC: Stained using FITC-conjugated goat anti-rat IgG; AF647: Stained using AF647-conjugated goat anti-mouse IgG; Merge: Superposition of different fluorescence images (Merge of images); (**B**) Immunoelectron microscopy (IEM) co-localization of EnSSB. (**a**–**f**) IEM co-localization of EnGAM22 and EnSSB in macrogametocytes incubated with rat anti-rEnGAM22 pAb (visualized with 18 nm gold particles) and mouse anti-rEnSSB pAb (visualized with 10 nm gold particles); (**a**,**c**) Early-stage macrogametocytes showing multiple WFB1 and WFB2 dispersed throughout the cytoplasm; (**b**,**d**) Enlarged sections of (**a**,**c**) reveal that EnGAM22 was located in WFB1 (▲) while EnSSB was located in cytoplasm (△); (**e**) A slightly later stage of macrogametocytes with initially formed OW and WFB2; **f** Enlarged sections of (**e**) showing that EnGAM22 was located in OW (▲) while EnSSB remained in cytoplasm (△). WFB1: Types 1 wall-forming bodies; WFB2: Types 2 wall-forming bodies; OW: Outer layer of oocyst wall.

To determine the subcellular localization of native EnSSB and assess whether EnSSB directly participated in oocyst wall formation, *E. necatrix* macrogametocytes in tissue samples were analyzed using IFA with mouse anti-rEnSSB pAb and rat anti-rEnGAM22 pAb or rat anti-rEnGAM59 pAb as first antibodies, and using AF647-conjugated goat anti-mouse IgG (red fluorescence) and FITC-conjugated goat anti-rat IgG (green fluorescence) as second antibodies. Meanwhile, the tissue sections were stained with haematoxylin and eosin to confirm the developmental stages of parasites. The results demonstrated that EnSSB did not co-localize with both of EnGAM22 and EnGAM59, whereas distributed in the cytoplasm and presented as granular form (Figure 5A).

The ultrastructural localization of native EnSSB in the mature macrogametocytes and formation of oocyst walls of *E. necatrix* were examined using immuno-electron microscopy (IEM). Tissue samples were co-labeled with the mouse anti-rEnSSB pAb and rat anti-rEnGAM22 pAb together, and then visualized using goat anti-mouse IgG conjugated to 10 nm golden particles and goat anti-rat IgG conjugated to 18 nm golden particles. The results showed that WFB1 in the mature macrogametocytes was specifically labeled with 18 nm gold particles (EnGAM22), whereas the 10 nm gold particles (EnSSB) distributed in the cytoplasm (Figure 5B). As the fusion of the WFB1 at the surface of the parasite and the release of their contents, the outer layer of the oocyst wall was formed. By this time, the 18 nm gold particles were specifically distributed in the outer layer, while the 10 nm gold particles remained in the cytoplasm (Figure 5B), which indicated that EnSSB did not directly participate in oocyst wall formation of *E. necatrix*.

### 3.6. Protective Efficacy of Vaccination on E. necatrix Challenge

Protective efficacy of rEnSSB was described in Table 3. No chicken died from coccidian challenge in any group in this study. BWG of UC and IC-L groups but not IC-H and IC-M groups were significantly reduced compared with UU group (*p* < 0.05). Among all immunization groups, RBWG of IC-M groups was the highest, but ROP of IC-H groups were the highest. Lesion score of IC-M groups was significantly reduced compared with UU group (*p* < 0.05). Groups (IC-M) of chickens immunized with 200 μg rEnSSB proteins resulted in ACI more than 160.

### 3.7. Serum Antibody Levels Against rEnSSB

**Figure 6:**
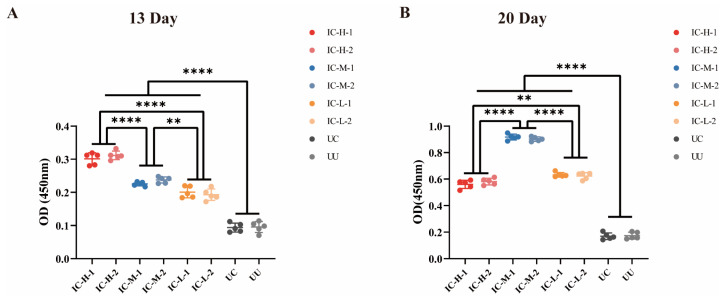
The antibody levels in the serum post the first immunization (**A**) and booster immunization (**B**). IC-H: immunized with high-dose of rEnSSB and challenged with oocysts group; IC-M: immunized with middle-dose of rEnSSB and challenged with oocysts group; IC-L: immunized with low-dose of rEnSSB and challenged with oocysts group; UC: unimmunized and challenged with oocysts group; UU: unimmunized and unchallenged with oocysts group. ** *p* < 0.01, **** *p* < 0.0001.

**Figure 7:**
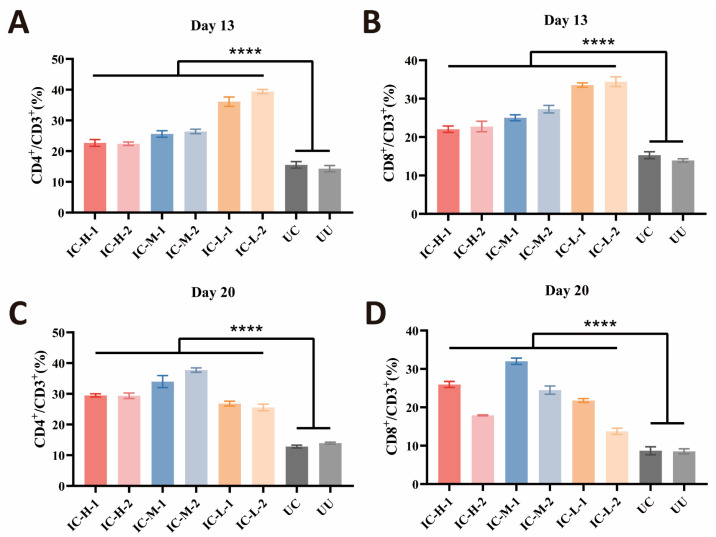
Proportion of T lymphocytes subpopulation in the immunized chicken spleen. (**A**) Proportion of CD4^+^/CD3^+^ T lymphocytes subpopulation post the first immunization. (**B**) Proportion of CD8^+^/CD3^+^ T lymphocytes subpopulation post the first immunization. (**C**) Proportion of CD4^+^/CD3^+^ T lymphocytes subpopulation post the booster immunization. (**D**) Proportion of CD8^+^/CD3^+^ T lymphocytes subpopulation post the booster immunization. IC-H: immunized with high-dose of rEnSSB and challenged with oocysts group; IC-M: immunized with middle-dose of rEnSSB and challenged with oocysts group; IC-L: immunized with low-dose of rEnSSB and challenged with oocysts group; UC: unimmunized and challenged with oocysts group; UU: unimmunized and unchallenged with oocysts group. **** *p* < 0.0001.

Serum IgY antibody level induced by rEnSSB in chickens were determined using indirect ELISA method. As depicted in Figure 6 and Table A1, one week after the first immunization and booster immunization, the IgY antibody titer induced by rEnSSB in immunized groups was significantly higher than that in UC and UU groups (*p* < 0.0001). One week post booster immunization, IgY antibody levels exhibited a sharp increase in the immunized groups, and the IgY antibody titer in IC-M groups being significantly higher than that in IC-H or IC-L groups (Figure 7B).

### 3.8. Effect of rEnSSB Immunization on CD4^+^/CD3^+^ and CD8^+^/CD3^+^ T Lymphocytes Subpopulation in Chickens

The effect of rEnSSB immunization on CD8^+^/CD3^+^ and CD4^+^/CD3^+^ T lymphocytes subpopulation in chicken spleen was assessed by flow cytometry. As depicted in Figure 7, one week after the primary and booster immunizations, rEnSSB immunization significantly increased the proportions of CD4^+^/CD3^+^ and CD8^+^/CD3^+^ splenic T lymphocytes compared to UC and UU groups (*p* < 0.0001). There is no significant difference between UC group and UU group (*p* > 0.05).

## 4. Discussion

SSB proteins are a class of highly conserved nucleic acid-associated proteins that play a pivotal role in maintaining genome integrity during replication, recombination, and repair by stabilizing single-stranded DNA intermediates [41]. In prokaryotes and eukaryotes, these proteins are characterized by a conserved oligonucleotide/oligosaccharide-binding (OB) fold domain that facilitates high-affinity interaction with single-stranded DNA. The OB-fold is a five-stranded β-barrel structure that forms a binding surface for ssDNA and is considered the signature motif of SSB family proteins across prokaryotes and eukaryotes [42]. In this study, the full-length cDNA of *ENH_00003220* gene, annotated as a hypothetical protein, was cloned a from *E. necatrix*. The cDNA is 1488 bp long and encodes a 495-aa protein with a predicted molecular weight of approximately 53.31 kDa. Sequence analysis revealed a conserved OB-fold SSB domain between residues 326 and 470, similar to SSB proteins from other organisms. The predicted tertiary structure also showed a typical OB-fold domain. Therefore, this protein is classified as a member of the SSB protein family and has been named EnSSB.

SSB proteins can be divided to three groups in accordance with their subunit structure: homotetramer, in which all subunits are encoded by the same gene, homodimer and heterotrimer of three different subunits [27]. Most prokaryotic SSBs and mitochondrial SSBs form homotetrameric structures. Eubacterial SSB monomer comprises two parts: the N-terminal DNA-binding domain with the OB-fold and the less structured C-terminal region containing conserved negatively charged amino acid residues [26]. Mitochondrial SSBs are homologues of eubacterial SSBs, but lack a long, negatively charged C-terminal tail [43]. In eukaryotes, RPA acts as an SSB protein and typically consists of a 70 kDa (RPA1), 32 kDa (RPA2) and 14 kDa (RPA3) subunits. The RPA1 in humans and yeasts possesses three distinct domains: an N-terminal protein interaction domain, a central ssDNA-binding area containing two OB-folds, and a C-terminal subunit-interacting region containing an evolutionarily conserved zinc finger motif [27]. In *C. fasciculata*, RPA is a 1:1:1 complex of three polypeptides of 51 kDa (CfaRPA1), 28 kD (CfaRPA2), and 14 kDa (CfaRPA3) [29]. In *C. parvum*, four RPA subunits have been identified, including two RPA1 subunits (~50 kDa CpRPA1A and ~70 kDa CpRPA1B), a single RPA2 subunit (~34 kDa), and a single RPA3 subunit (~12 kDa) [30,31,44,45]. In *P. falciparum*, a large subunit (PfRPA1) has been characterized, which also has only ~55 kDa [32]. These three protozoan RPA1 proteins (CfaRPA1, CpRPA1A and PfRPA1) lack the N-terminal protein–protein interaction sequence comparison to those of higher eukaryotes, and are all short-type subunits. In the present study, EnSSB protein was 495 aa in length with a theoretical molecular weight of 53.31 kDa, and its native protein with ~58 kDa in size was confirmed by Western blot analysis. The sequence analysis showed that EnSSB has only one OB-fold domain, and lacks the N-terminal protein-interaction domain and the C-terminal subunit-interacting region. Moreover, in the N-terminal part of EnSSB, no significant homologies to any known proteins were found; in the C-terminal part composed of 25 amino acids, there are 11 glycine residues and 10 arginine residues, indicating that EnSSB has a positively charged tail. In addition, EnSSB exhibited the closest evolutionary relationship with human mitochondrial SSB, RIM1 protein of yeast—encoded by a nuclear gene whose product participates in mtDNA metabolism [46], and PfSSB protein—encoded in the nucleus and targeted to the apicoplast of *P. falciparum* [47]. Based on the analysis, we propose that EnSSB is encoded in the nucleus and targeted to the mitochondria or the apicoplast of *E. necatrix*.

In a prior study [14], it was found that the EnSSB protein (GenBank: XP_013433731.1) exhibits differential expression between the oocyst wall and WFBs, with minimal expression in WFBs. To determine whether EnSSB directly participates in oocyst wall formation, we used IFA to examine its localization in mature *E. necatrix* macrogametes. We found that EnSSB is granularly distributed in the cytoplasm and does not co-localize with EnGAM22 or EnGAM59. Due to limitations in research methods, we did not perform quantitative analysis of the immunofluorescence images. This lack of quantification represents a limitation in the current study. In future work, we will use image quantification tools to enhance the rigor of our analysis. Furthermore, we used the immunogold electron microscopic co-localization technique to define the ultrastructural localization of EnSSB in the mature *E. necatrix* macrogametes. We found that EnSSB distributes in the cytoplasm, but not in the nucleus and developmental oocyst wall, and also does not co-localize with both of EnGAM22 and EnGAM59. These results, combined with subcellular localization predictions by UniProt and Cell-PLoc-2, further suggest that EnSSB is encoded in the nucleus and targeted to the mitochondria. Of course, this conclusion still needs to be verified through experiments, such as co-localization with mitochondrial proteins.

In Western blot analysis using anti-rEnSSB pAb, we detected the native EnSSB protein in protein extracts from MZ-2, GAM, and UO, but not in MZ-3. qPCR analysis revealed significantly higher EnSSB transcript levels in UO compared to MZ-2, MZ-3, and GAM, and higher levels in GAM than in MZ-2 and MZ-3. These results suggest that EnSSB may play a regulatory role in gametogony and sporogony rather than merogony. Interestingly, the native EnSSB protein had a size of approximately 58 kDa in MZ-2 and UO, which is close to the theoretical molecular mass of 53.1 kDa. However, apart from extremely weak band at ~58 kDa, the four bands of ~98, ~82, ~36 and ~28 kDa were detected in the extract prepared from GAM. The appearance of ~36 and ~28 kDa bands may be attributed to potential post-translational cleavage of the protein in the parasite, as described by Millership and Zhu [31]. While a minor band at 75 kDa, corresponding to the protein encoded by the *CpRPA1B* gene in *C. parvum* sporozoites was observed, the same blotting analysis also revealed a major band at 40–43 kDa. It must also be considered that EnSSB gene might be translated from an alternative initiation codon, as noted by Millership et al. [44], where despite the *CpRPA2* gene predicting a 40.1 kDa peptide, Western blot analysis of oocyst preparations identified a native CpRPA2 protein with a molecular mass of approximately 32 kDa. Sequence analysis revealed that the OB-fold domain of EnSSB is located between amino acid residues 326 and 470, and the first 765 nucleotides (255 amino acids) of the putative ORF of the EnSSB gene contain four methionine codons. If EnSSB protein translation starts from the fourth methionine codon, its theoretical molecular weight is 26.2 kDa, which is near the range of ~28 kDa. Both possibilities require further molecular and proteomic analyses to reach a firm conclusion. While we acknowledge the significance of using proteomics to validate our conclusions, it was not applied in this experiment. Future studies will utilize proteomics methods to investigate this possibility and confirm the underlying mechanisms of these observations.

Additionally, there are also two distinct bands in the extract prepared from GAM that are significantly larger than ~58 kDa. It is known that sodium dodecyl sulfate (SDS), an anionic detergent, disrupts the natural structure of proteins. In standard SDS-PAGE, multimeric proteins dissociate into monomers and cannot maintain their polymeric form. Therefore, the observation likely resulted from proteins in GAM that cross-react with anti-rEnSSB pAb and have a larger molecular weight than EnSSB. As described by Jong et al. [48], antibodies to SSB-1 of *S. cerevisiae* also react with three other proteins in extracts: a 37 kDa protein, possibly a proteolytic product, and two other species of 75 and 55 kDa, which may be precursors or distinct but immunologically related gene products. Further investigations, such as mass spectrometry, will be necessary to clarify the nature of these protein molecules. On the other hand, the gametocyte is a crucial developmental phase in the life cycle of *Eimeria* parasites, involving sexual differentiation, fertilization, and oocyst formation. It is possible that the protein complex formed by EnSSB and its interacting proteins remained intact during SDS-PAGE and was thus detected. The presence of EnSSB of varying sizes in gametocytes may indicate they play specialized roles in DNA metabolism and genome stability during gametogenesis.

SSB protein plays a crucial role in DNA metabolism and should be an important target antigen for vaccine development. Unfortunately, there are very few studies on the immune-protective function of SSB protein. Recently, Wu et al. [49] found that the SSB protein (ICP8) of herpesviruses is a prime target for studying the pathogenicity of the duck plague virus and for vaccine development. A mutation at amino acid C514 of ICP8 decreases ssDNA binding, reduces DPV virulence, and provides strong protection against lethal challenges. In this study, rEnSSB demonstrated effective immune protection against coccidia challenge. It significantly reduced intestinal damage, oocyst shedding, and weight loss. Chickens immunized with 100 μg of rEnSSB had an ACI of 165.16. The immunization triggered humoral immunity and antibody production, and increased the proportions of CD4^+^ and CD8^+^ T lymphocytes. These findings suggest that EnSSB can be used to develop a recombinant coccidiosis vaccine.

## 5. Conclusions

In conclusion, we have cloned and sequenced the cDNA of a hypothetical protein (NCBI: XP_013433731.1) from *E. necatrix*, confirming it as the *E. necatrix* SSB gene (EnSSB). The EnSSB gene encodes a 53.1 kDa peptide with a conserved OB-fold domain, lacking the N-terminal protein-interaction domain and the C-terminal subunit-interacting region. Phylogenetic tree analysis and subcellular localization studies using software prediction, IFA, and immunoelectron microscopy suggest that EnSSB is encoded in the nucleus and targeted to the mitochondria of *E. necatrix*. Chickens immunized with rEnSSB protein performed better than unimmunized challenged chickens.

## Figures and Tables

**Table 1:** Sequences of primers.

Primer Name	Sequence 5′-3′
EnSSB-F	ATGGCGGAGTCCTTCAC
EnSSB-R	TTATATGCCTCTTCTCCCTCG
pET-EnSSB-F	ggaattccatatgATGGCGGAGTCCTTCAC
pET-EnSSB-R	cgcggatccTTATATGCCTCTTCTCCCTCG
qEnSSB-F	CATACAGCAGGTCAAGCCAGAGATC
qEnSSB-R	CTGAGTTCCGCAGCACGATGAG
pMD-18T-RV-M	AGCGGATAACAATTTCACACAGGA
pMD-18T-M13-47	CGCCAGGGTTTTCCCAGTCACGAC
pET-28a-T7	TAATACGACTCACTATAGGG
pET-28a-T7ter	TGCTAGTTATTGCTCAGCGG
qEn18S-F	GAAACTGCGAATGGCTCATT
qEn18S-R	CTTGCGCGTACTAGGCATTC

**Table 2:** Details of the immunization schedule employed in the vaccination/challenge study.

Groups	Names	No. of Chickens	Immunization-Dose (μg) and Route		Challenge-Oocyst Dose (×10^4^) and Route
			6 Days Old *	13 Days Old **	20 Days Old
1	IC-H-1	20	200/S.C ***	200/S.C	2/Oral gavage
2	IC-H-2	20	200/S.C	200/S.C	2/Oral gavage
3	IC-M-1	20	100/S.C	100/S.C	2/Oral gavage
4	IC-M-2	20	100/S.C	100/S.C	2/Oral gavage
5	IC-L-1	20	50/S.C	50/S.C	2/Oral gavage
6	IC-L-2	20	50/S.C	50/S.C	2/Oral gavage
7	UC	20	PBS/S.C	PBS/S.C	2/Oral gavage
8	UU	20	PBS/S.C	PBS/S.C	PBS/Oral gavage

*: rEnSSB protein emulsified with Freund’s complete adjuvant (FCA; Sigma); **: rEnSSB protein emulsified with Freund’s incomplete adjuvant (FIA; Sigma); S.C route ***: Subcutaneous injection route.

**Table 3:** Protective efficacy of rEnSSB protein against *E. necatrix* challenge.

Groups	Names	SR (%)	BWG (g)	RBWG (%)	OP Per Bird (×10^6^)	ROP (%)	LS	LI	OI	ACI
1	IC-H-1	100	47.99 ± 8.39 ^abc^	82.94	0.92	71.25	2.30 ± 0.54 ^bcd^	23.00	10.00	149.94
2	IC-H-2	100	47.29 ± 8.33 ^abc^	81.73	0.94	70.63	2.15 ± 0.58 ^bcd^	21.50	10.00	150.23
3	IC-M-1	100	54.48 ± 9.99 ^c^	94.16	1.53	52.19	1.90 ± 0.52 ^b^	19.00	10.00	165.16
4	IC-M-2	100	54.19 ± 8.92 ^bc^	93.66	1.35	57.81	1.85 ± 0.67 ^b^	18.50	10.00	165.16
5	IC-L-1	100	39.33 ± 14.09 ^a^	67.97	2.38	25.63	2.65 ± 0.41 ^cd^	26.50	20.00	121.47
6	IC-L-2	100	42.38 ± 15.63 ^a^	73.25	2.17	32.19	2.55 ± 0.64 ^bcd^	25.50	20.00	127.75
7	UC	100	43.93 ± 11.50 ^a^	75.92	3.20	0.00	2.90 ± 0.39 ^d^	29.00	40.00	106.92
8	UU	100	57.86 ± 8.82 ^bc^	100.00	NA	100.00	0.00 ± 0.00 ^a^	0.00	0.00	200.00

Note: SR: survival rate; BWG: body weight gain; RBWG: relative body weight gain; ROP: reduction in oocyst production; OP: oocyst production; LS: lesion score; LI: lesion index; OI: oocyst index; ACI: anticoccidial index; IC-H: immunized with high-dose of rEnSSB and challenged with oocysts group; IC-M: immunized with middle-dose of rEnSSB and challenged with oocysts group; IC-L: immunized with low-dose of rEnSSB and challenged with oocysts group; UC: unimmunized and challenged with oocysts group; UU: unimmunized and unchallenged with oocysts group. Oocyst output of Group 8 (UU) was zero and shown as “NA”. Different superscript letters (a, b, c, d) denote statistically significant differences between groups at *p* < 0.05.

## Data Availability

The raw data supporting the conclusions of this article will be made available by the authors on request.

## Appendix A

**Table A1:** The mean optical density and values along with the standard deviation (mean OD ± SD) of antibody levels in the serum post the first immunization (13 Day) and booster immunization (20 Day).

Group	IC-H-1	IC-H-2	IC-M-1	IC-M-2	IC-L-1	IC-L-2	UC	UU
13 Day	0.3012 ± 0.01824	0.3116 ± 0.01299	0.2254 ± 0.006066	0.2374 ± 0.009370	0.2010 ± 0.01749	0.1930 ± 0.01718	0.09380 ± 0.01352	0.09540 ± 0.01673
20 Day	0.5604 ± 0.03109	0.5816 ± 0.02503	0.9174 ± 0.02182	0.9018 ± 0.01663	0.6324 ± 0.01704	0.6232 ± 0.02567	0.1690 ± 0.02481	0.1736 ± 0.02586
